# Microneedles as Modern Carriers of Plant Extracts

**DOI:** 10.3390/mi16020143

**Published:** 2025-01-26

**Authors:** Jagoda Chudzińska-Skorupinska, Agata Wawrzyńczak, Agnieszka Feliczak-Guzik

**Affiliations:** Department of Applied Chemistry, Faculty of Chemistry, Adam Mickiewicz University in Poznań, Uniwersytetu Poznańskiego 8, 61-614 Poznań, Poland; jagoda.chudzinska@amu.edu.pl (J.C.-S.); agata.wawrzynczak@amu.edu.pl (A.W.)

**Keywords:** microneedles, sodium hyaluronate, red beet extract, parsley leaf extract, apigenin, betanin

## Abstract

Recently, intensive research has been conducted on effective and simple systems for delivering active substances deep into the epidermis, e.g., for the treatment of skin inflammation. One possibility can be the use of soluble microneedles in which active compounds are encapsulated. This article describes the preparation of modern carriers, namely microneedles with encapsulated extracts of red beet or parsley leaves, that are rich in active substances with antioxidant and anti-inflammatory properties, specifically betanin and apigenin. The concentration of hyaluronic acid sodium salt, the method of preparing the solution, and the technique of the complete filling of molds were optimized. Plant extracts were obtained with sonication or maceration. In order to characterize the extracts obtained, several techniques were employed, such as UV–Vis, LC–MS, GC–MS, and FTIR-ATR. The analyses performed allowed for confirmation of the presence of selected active substances in the extracts. The most optimal solution of the microneedles’ precursor turned out to be the one with a concentration of 10 wt.% of sodium hyaluronate, prepared by stirring and sonication. The most efficient extraction method for each plant was chosen, and the extracts were introduced into a solution of hyaluronic acid sodium salt. The resulting soluble microneedle patches can be used as an alternative to the traditional methods of delivering anti-inflammatory and antioxidant substances of plant origin.

## 1. Introduction

Microneedle patches are a good alternative to reduce production costs and simplify the process of synthesizing novel transdermal carriers. Microneedles are small spears up to 2000 μm long that penetrate the skin to deliver active substances directly to a specific layer of the epidermis, depending on the length of the needles [1]. These carriers can be created from many materials, including ceramic, glass, metal, silicone, or polymers. In addition, they have different structures, on which the release of active substances depends. Among them, solid, coated, hollow, hydrogel-based, or spreadable microneedles can be distinguished [2]. In particular, soluble microneedles are gaining increasing popularity. They are most often prepared on the basis of dissolvable polymers, such as natural polysaccharides, e.g., hyaluronic acid [3,4]. The main advantage of microneedle patches is the simplicity of their synthesis and ease of application [5]. In addition, small spears based on natural dissolvable polymers generate negligible pain sensation compared to traditional injections, as well as minimize waste that is difficult to dispose of, which is in line with the principles of sustainability [6,7]. Dissolvable microneedles can be designed to release the active ingredient in a specific time-dependent manner, based on rapid or slow dissolution [8]. An advantage of this type of carrier is also the ability to encapsulate a wide range of active substances. Currently, as a result of the ever-growing trend of naturalness, active substances of plant origin are in demand.

Plants are rich in various types of active substances that exhibit desirable biological activity with anti-inflammatory, antioxidant, and anticancer properties [9]. Plants rich in phenolic compounds, including flavonoids, with the properties mentioned above are red beet (*Beta vulgaris* L.) and parsley leaves (*Petroselinum crispum* L.), widely grown in Europe [10,11]. Red beet is characterized by its content of betalains, which are water-soluble pigments that include nitrogen atoms in their molecules. Among them, betacyanins can be distinguished, which are responsible for the characteristic red-purple color. These include betanin, prebetanin, and isobetanin. Betalains also include a group of betaxanthin compounds of a yellow-orange color [12,13]. In addition, red beet is rich in flavonoids, including, among others, betagarin [12,14]. All these substances are primarily responsible for the anti-inflammatory, antioxidant, and anticancer properties of this plant [15]. Parsley leaves are also rich in biologically active compounds, mainly polyphenolic compounds such as quercetin, apigenin, myricetin, and p-coumaric acid, some of which may be in the form of glycosides [16,17,18]. These compounds give parsley, which for many years has been widely used as an herb, antioxidant properties [19,20].

Extracts of red beet and parsley leaves can be used successfully, for example, to prevent inflammatory changes in the skin, because they contain many active compounds with anti-inflammatory and antioxidant properties. However, to effectively deliver active substances deep into the epidermis, it is necessary to encapsulate them in appropriate carriers that support transdermal transport [21]. According to the recent literature reports, red beet extract has been encapsulated in chitosan nanocomposites (BR@CS NC), chitosan-based nanogel, and nano-liposomal systems or PEGylated gelatin nanoparticles [22,23,24,25]. On the other hand, parsley leaf extract has been used so far in the syntheses of silver and gold nanoparticles, as well as in colloidal core–shell nanocomposites with antibacterial, antioxidant, and anticancer properties [26,27,28]. Nevertheless, obtaining the carriers mentioned above is usually complicated, demanding, and expensive.

Dissolvable microneedle patches based on hyaluronic acid sodium salt can be excellent transdermal carriers for red beet and parsley leaf extracts due to their low production costs and negligible waste production, which is in line with the trend of sustainable development. These carriers containing the above-mentioned plant extracts, due to the presence of active compounds of an anti-inflammatory and antioxidant nature, can be used to soothe inflammation of the skin. In addition, it is worth mentioning that dissolvable microneedle patches have not yet been reported in the literature as a delivery system for actives extracted from red beet and parsley leaves.

## 2. Materials and Methods

### 2.1. Extracts

#### 2.1.1. Preparation of the Extracts

**Figure 1:**
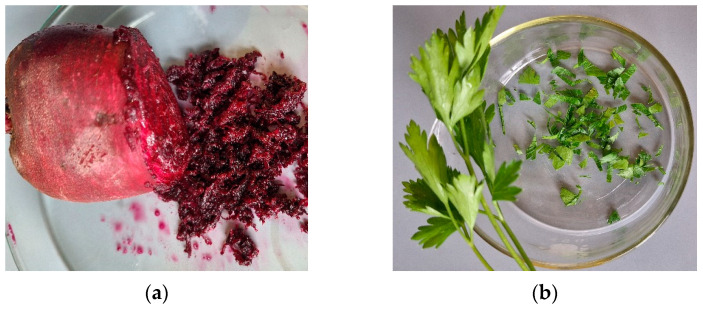
Plant material used for extraction: (**a**) red beet (*Beta vulgaris* L.); (**b**) parsley leaves (*Petroselinum crispum* L.).

The extracts were prepared using red beet (*Beta vulgaris* L.) and parsley leaves (*Petroselinum crispum* L.) from a local field in the Wielkopolska region (Poland). After washing with warm water, a red beet without previous peeling was ground on a vegetable grater and the parsley leaves were cut manually into smaller pieces (Figure 1).

The plant material was placed in glass bottles and ethanol (Stanlab, 96%) was added in a weight ratio of 1:2 (red beet) and 1:4 (parsley leaves). The bottles were then tightly sealed, and the plant material was (i) macerated for 24 h, or (ii) subjected to sonication for 1 h (37 kHz), then stirred on a magnetic stirrer for 3 h (500 rpm) and again subjected to ultrasound (37 kHz) for 1 h, resulting in a total of 5 h of ultrasound-assisted extraction.

When the extraction was complete, each sample was filtered using quantitative filter papers (type 391—very fine pores, weight: 84 g/m^2^) to separate the spent plant material. Before analysis, each sample was subjected to additional filtration through a syringe filter (PTFE; pores: 0.22 μm; hydrophilic).

#### 2.1.2. UV–Vis Analysis

For qualitative analysis of the prepared plant extracts, measurements using UV–Vis spectroscopy (Nicolet Evolution 220 PC spectrophotometer, measurement range 200–800 nm) were performed. In addition, the standards of the active substances present in the tested plant extracts, i.e., betanin and apigenin, were analyzed. The characteristic wavelengths corresponding to the maximum absorbance of the active substances used were as follows: (a) betanin: 536 nm; (b) apigenin: 268 nm.

#### 2.1.3. LC–MS Analysis

LC–MS analysis of the selected active substances, namely betanin and apigenin, as well as plant extracts obtained from red beet and parsley leaves was performed. LC–MS spectra were collected for extract samples obtained after 24 h of maceration (sample name: X*_M_24h, where *X = B for red beet extract and *X = P for parsley leaf extract) and with ultrasound-assisted extraction (sample name: X*_U_5h, where *X = B for red beet extract, and *X = P for parsley leaf extract).

An SQD-2 mass spectrometer was used for LC–MS analysis. ESI-MS spectra were recorded in the mass range of 100–1000. Water was used as a solvent and the analysis time was 60 min. All samples, namely, the standards for betanin and apigenin as well as plant extracts, were analyzed following the same procedure.

#### 2.1.4. GC–MS Analysis

In order to qualitatively analyze the obtained plant extracts and compare the selected extraction methods, GC–MS analysis of the samples was performed after 5 h (X_M_5h) and 24 h of maceration (X_M_24h), as well as after finishing the ultrasound treatment (X_U_5h).

Analyses were performed using a Varian GC–MS 4000 (Palo Alto, CA, USA) apparatus equipped with an automatic sample feeder. Helium was used as the carrier gas, with a flow rate of 1 mL/min. The total analysis time was 60 min.

Based on the results, the most efficient extraction method was selected, and the chosen extract was then introduced into a prepared microneedle patch.

#### 2.1.5. FTIR-ATR Analysis

In order to evaluate the presence of standard substances (betanin and apigenin) in the resulting plant extracts, FTIR-ATR analysis was carried out with a Nicolet iS50 FTIR spectrophotometer (Thermo Fisher Scientific, Waltham, MA, USA), which operated in the attenuated total reflection mode. All samples were tested in the range of 4000 cm^−1^ to 650 cm^−1^. The apparatus had a resolution of 16 cm^−1^ with a spectral scan of 64 times for the sample and 8 times for the background.

#### 2.1.6. Antioxidant Activity Test

Antioxidant activity tests were performed using the free radical DPPH (2,2-di(4-tert-octylphenyl)-1-picrylhydrazyl, free radical, Sigma-Aldrich, St. Louis, MO, USA). For this purpose, 3.03 mg of DPPH was dissolved in 100 mL of ethanol, and spectrophotometric measurements were carried out to establish the wavelength (λ_max_) at which the absorbance of the DPPH radical was the highest (Nicolet Evolution 220 PC spectrophotometer, measurement range 200–800 nm). Then, 1.5 mL of the DPPH radical solution and 1.5 mL of the sample were combined into a quartz cuvette. During the first 10 min, the absorbance was measured every minute and then additional measurements were made 15 min, 20 min, 25 min, 45 min, and 60 min after mixing the test sample and the DPPH solution. The antioxidant activity of selected extracts and standard substances, namely apigenin and betanin, was analyzed. The percentage of free radical quenching was calculated according to Equation (1):

% of inhibition = ((A_0_ − A_1_)/A_0_)/100 (1)

A_0_—absorbance of the radical solution [a.u.];

A_1_—absorbance of the radical solution after the addition of the test sample [a.u.].

### 2.2. Microneedles

#### 2.2.1. Silicone Mold Preparation

In order to prepare a silicone mold for the casting of the microneedle patch, a polymer mold with the following dimensions was used:

- Patch size: 8 mm × 8 mm;
- Array size: 10 × 10 needles;
- Needle height: 1 mm;
- Needle base: 0.2 mm;
- Needle pitch (distance between needles): 0.5 mm.

The mold was prepared with 3D printing (PolyJet), using liquid photopolymers hardened with UV light.

A solution of polydimethylsiloxane (PDMS, Sylgard 184, Dow, Midland, MI, USA) was prepared by mixing the PDMS monomer and the cross-linking agent in a 10:1 ratio. The PDMS solution was poured into a polymer mold and allowed to polymerize for 24 h. The finished silicone mold was used to prepare microneedle patches after complete optimization of the preparation procedure for the solution of hyaluronic acid sodium salt.

#### 2.2.2. Preparation of the Microneedle Patch

Optimization of the preparation procedure was performed on “empty” patches. A microneedle patch was prepared using hyaluronic acid sodium salt (molecular weight: 30,000–50,000 Da, Chemat, Gdańsk, Poland) and a plant extract was prepared using the selected method.

Optimization of the selection of the concentration of hyaluronic acid sodium salt was carried out. For this purpose, a set of solutions was prepared at 5 wt.%, 10 wt.%, 15 wt.%, 20 wt.%, and 25 wt.%. An appropriate amount of hyaluronic acid sodium salt was dissolved in distilled water by topping up to 100 wt.%. On the basis of the organoleptic observation of the density of the prepared solutions, the most optimal concentration of hyaluronic acid sodium salt was selected.

The selection of the best method for the dissolution of the hyaluronic acid sodium salt and the degassing of the solution obtained was carried out using the following:

- Ultrasonic bath (37 kHz, 45 min);
- Shaker (300 rpm, 80 min);
- Manual stirring and ultrasonic bath (2 min; 37 kHz, 25 min);
- Manual stirring and shaker (2 min; 300 rpm, 85 min).

After selecting the most optimal method to prepare the hyaluronic acid sodium salt solution, the procedure for the complete filling of the silicone mold was also optimized by applying the following processes:

- Direct pouring into the mold;
- Sonication (2 min; 37 kHz);
- Shaker (5 min; 300 rpm);
- Freezing (three cycles of freezing at −20 °C for 30 min and thawing at room temperature of approximately 23 °C for 30 min; in total 3 h).

During the optimization procedures mentioned above, a substitute (Figure S1, Supplementary Materials) of the actual silicone mold was used. It had the form of a square with a size and shape that mimicked the target mold of the microneedle patch.

Optimization of the procedure for obtaining microneedle patches incorporated with plant extracts was also performed. After selecting the most optimal method to obtain the polymer base, a 10 wt.% solution of hyaluronic acid sodium salt was prepared with 1 wt.% of the selected extract added. The solution was stirred manually for approximately 2 min and then subjected to sonication (37 kHz) for 25 min in order to degas the solution. After this time, the prepared solution was poured into silicone molds. The molds with the solution were subjected to a freezing and thawing procedure to completely fill the molds and remove air bubbles. The molds with the solution were then left to evaporate and air dry at room temperature for 24 h. After this time, the finished microneedle patches were removed from the molds.

#### 2.2.3. Stereoscopic Microscope

Microscopic analysis of the microneedle patches prepared with hyaluronic acid salt solutions with different concentrations was carried out to determine the shape of the resulting microneedles. The analysis was carried out using a StereoLumar V12 stereoscopic microscope (Zeiss AG, Oberkochen, Germany).

#### 2.2.4. Release Profiles of Active Substance from Microneedles

The release profile of the active substance from the prepared carriers was studied by measuring the absorbance (Nicolet Evolution 220 PC spectrophotometer, measurement range 200–800 nm) of the solutions tested at characteristic wavelengths corresponding to the maximum absorbance of the active substances, namely betanin (λ_max_ = 536 nm) and apigenin (λ_max_ = 268 nm). The release tests were carried out in duplicate, using 5.0 mL of phosphate buffer (pH = 5.8). Then, the results were averaged, and the standard deviation was calculated.

The study of betanin release from the carrier containing red beet extract was performed by measuring the absorbance 1 min, 2 min, 3 min, 5 min, and 10 min after the sample was combined with the solvent. On the other hand, the study of apigenin release from the carrier containing parsley leaf extract was carried out by measuring the absorbance every minute for a period of 11 min and then after 15 min. Between measurements, the sample solution with the acceptor medium was stirred with a magnetic stirrer (rpm = 400). Based on the standard curves created (Figure S7 and Figure S8), the concentrations of active substances were calculated.

## 3. Results

### 3.1. Extracts

#### 3.1.1. UV-Vis

**Figure 2:**
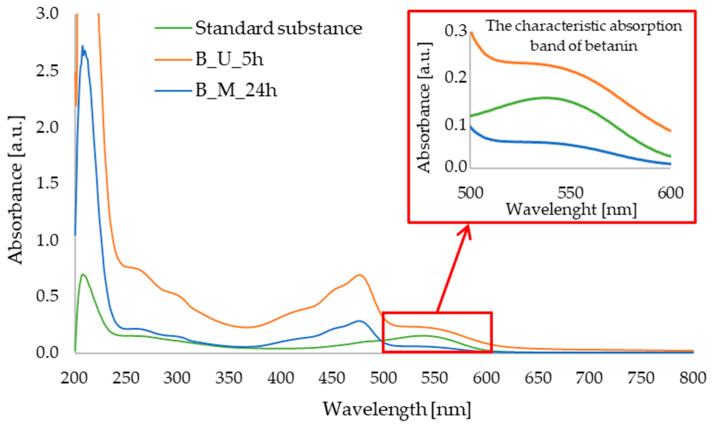
UV–Vis spectra for: standard substance (betanin); red beet extract obtained after 5 h of ultrasound-assisted extraction (B_U_5h); red beet extract obtained after 24 h of maceration (B_M_24h).

Qualitative UV–Vis analysis allowed us to confirm the presence of betanin in the red beet extracts obtained. In Figure 2, an absorption band can be observed at a wavelength of approximately 538 nm for the standard substance and the red beet extract obtained using the ultrasound-assisted method. On the other hand, this band for the extract obtained with 24 h of maceration is not very intense. The maximum absorption of betanin has already been determined by the research group of Xu [29] and Yang [30], among others.

**Figure 3:**
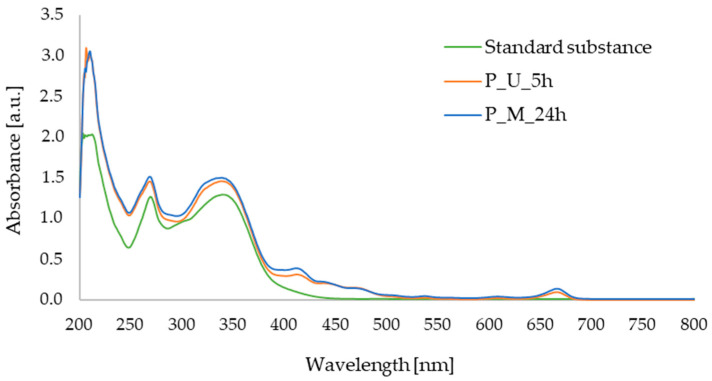
UV–Vis spectra for: standard substance (apigenin); parsley leaf extract obtained after 5 h of ultrasound-assisted extraction (P_U_5h); parsley leaf extract obtained after 24 h of maceration (P_M_24h).

In Figure 3, showing the UV–Vis spectra of apigenin and parsley leaf extract obtained using sonication or maceration procedures, two characteristic absorption maxima can be observed for the apigenin standard, namely at 339 nm and 269 nm. The same characteristic bands are observed in the spectra of the extracts obtained, indicating the presence of apigenin in the samples under study. The characteristic absorption bands of apigenin have previously been determined in the literature, for example, in a study conducted by a research group of Mevada et al., where the maximum absorption was at 268 nm [31], whereas Poureini et al. observed two absorption maxima for apigenin at 269 nm and 337 nm [20], which is in good agreement with our results.

#### 3.1.2. LC–MS

**Figure 4:**
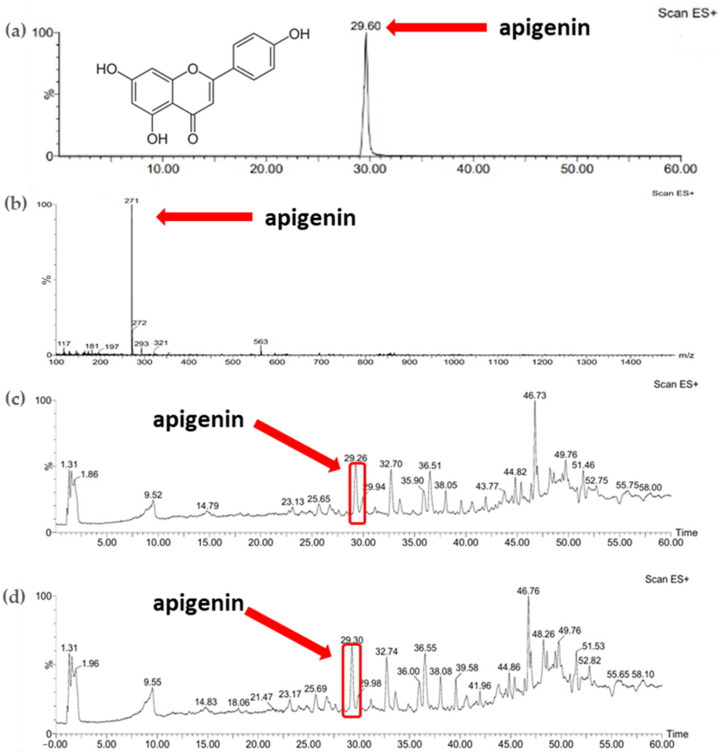
(**a**) Chromatogram of standard substance—apigenin; (**b**) mass spectrum of standard substance—apigenin; (**c**) chromatogram of parsley leaf extract obtained with sonication (P_U_5h); (**d**) chromatogram of parsley leaf extract obtained with 24 h of maceration (P_M_24h).

Comparison of the results obtained for the standards of active substances, namely apigenin and betanin, allowed the identification of these compounds in the plant extracts obtained. Figure 4b shows the ESI+/MS spectrum for the apigenin standard (apigenin mass: 270.24 Da). A pseudomolecular ion ([M-H]^+^) with a *m*/*z* value of 271 was observed and the retention time for the apigenin standard was approximately 30 min (Figure 4a). Based on the comparison of the retention time of apigenin in standard solution, the presence of the active substance was confirmed in the parsley extracts obtained using both extraction methods (Figure 4c,d).

**Figure 5:**
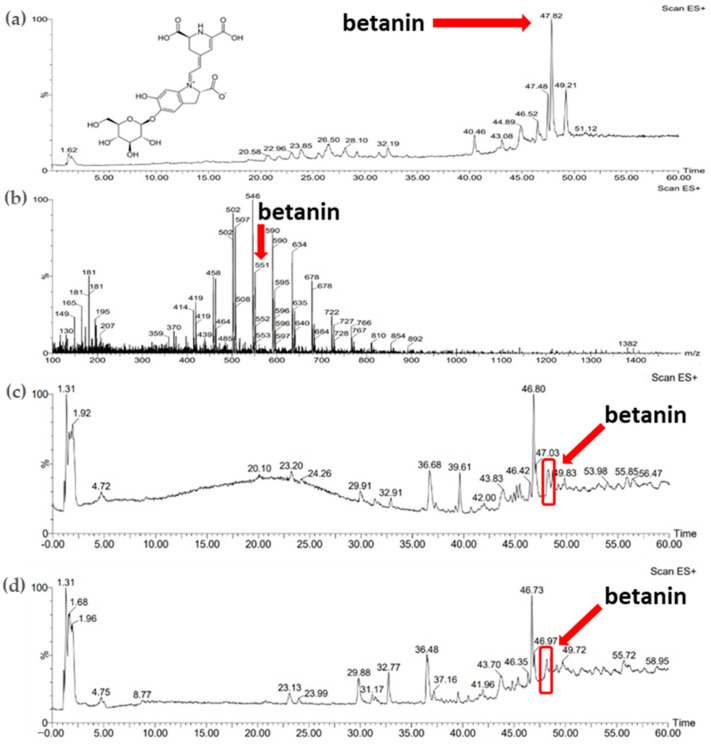
(**a**) Chromatogram of standard substance—betanin; (**b**) mass spectrum of standard substance—betanin; (**c**) chromatogram of red beet extract obtained with sonication (B_U_5h); (**d**) chromatogram of red beet extract obtained with 24 h of maceration (B_M_24h).

Figure 5b shows the ESI+/MS spectrum for the betanin standard (betanin mass: 550.49 Da). A pseudomolecular ion ([M-H]^+^) with a *m*/*z* value of 551 was observed (Figure 5b). The retention time of this compound determined from the chromatogram (Figure 5a) was approximately 47 min. Comparison of the retention time of the betanin standard with the chromatograms of plant extracts allowed the identification of betanin in the samples studied obtained using both extraction methods (Figure 5c,d).

#### 3.1.3. GC–MS

GC–MS analyses of the samples obtained allowed comparison of the compound profiles in the plant extracts obtained using two diverse extraction methods and different maceration times, namely 5 h (M-5H) and 24 h (M_24H). Table 1 summarizes all compounds identified using the GC–MS method in the samples obtained from red beet extracts. GC–MS analysis of red beetroot powder was carried out, among others, by a research group of Fiadorwu et al., which identified, for example, n-hexadecanoic acid [32], which coincides with the results obtained during our study.

GC–MS analysis also allowed for the identification of compounds present in parsley leaf extracts obtained using various extraction methods. On the basis of the mass spectra obtained, compounds present in the parsley leaf extracts were identified, as shown in Table 2. A similar study was carried out by a research group of Craft et al. [33], where α-pinene, myristicin, and β-myrcene, among others, were also determined.

#### 3.1.4. FTIR-ATR

**Figure 6:**
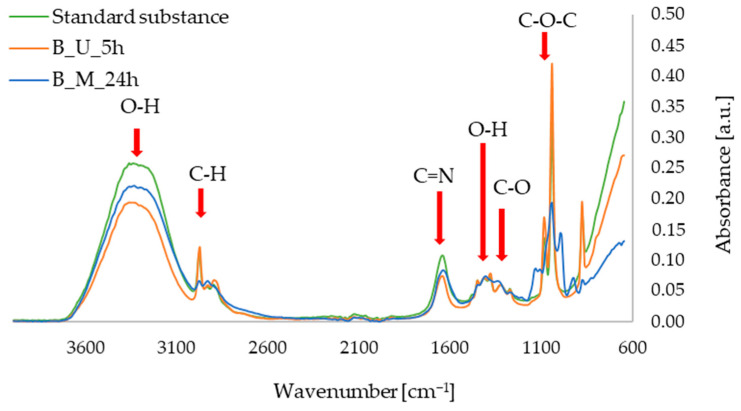
FTIR-ATR spectra for standard sample of betanin; red beet extract obtained with ultrasound-assisted procedure (B_U_5h); red beet extract obtained with 24 h of maceration (B_M_24h).

The FTIR-ATR measurements allowed us to obtain spectra of the active substances in the form of standards in ethanolic solutions, as well as the extracts of red beet and parsley leaves prepared with maceration and ultrasound-assisted procedures. In Figure 6, showing the FTIR-ATR spectra for the betanin and red beet extracts, characteristic bands can be observed, namely in the ranges of: 3600–3100 cm^−1^ (O–H alcohols, stretching vibrations), 3000–2900 cm^−1^ (C–H, stretching vibrations), 1700–1600 cm^−1^ (C=N, stretching vibrations), 1500–1350 cm^−1^ (O–H, deformation vibrations), 1350–1250 cm^−1^ (C–O, stretching vibrations), and 1100–1000 cm^−1^ (C–O–C, stretching vibrations). These vibrations correspond to the bonds found in the betanin molecule, as confirmed in the literature, including studies conducted by an Aztatzi-Rugerio research group [34], Barkociová et al. [35] and Mocanu et al. [36].

**Figure 7:**
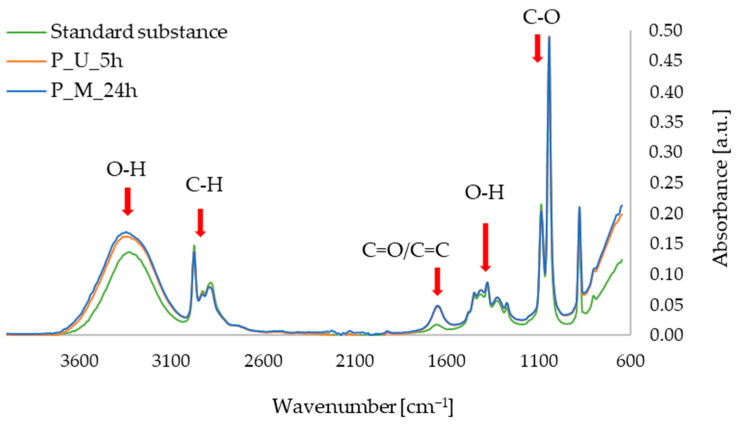
FTIR-ATR spectra for standard sample of apigenin; parsley leaf extract obtained with ultrasound-assisted procedure (P_U_5h); parsley leaf extract obtained with 24 h of maceration (P_M_24h).

In Figure 7, showing the FTIR-ATR spectra for apigenin and extracts from parsley leaves, characteristic bands can be seen in the range of 3600–3000 cm^−1^ (O–H alcohols, stretching vibrations), 3000–2850 cm^−1^ (C–H aliphatics, stretching vibrations), 1700–1600 cm^−1^ (C=O or C=C, stretching vibrations), 1500–1250 cm^−1^ (O–H, deformational vibrations), and 1100–1000 cm^−1^ (C–O, stretching vibrations). The characteristic oscillations listed above for apigenin (especially the oscillations at approximately 1650 cm^−1^) have previously been described in the literature, among others, in 2021 by the Amini research group [37], as well as by researchers led by Aldawsari [38].

#### 3.1.5. Antioxidant Activity Test

The study of antioxidant activity allowed for the determination of the antioxidant properties of the extracts obtained and the active substances present in them. Figure S9 shows the dependence between absorbance and quenching time for the standard of betanin and beet extract obtained with the ultrasound-assisted method. The free radical quenching for the standard substance is 20.02%, while that of the tested extract is 19.75%.

Figure S10 shows a plot of the dependence between absorbance and quenching time for apigenin and parsley leaf extract obtained with 24 h maceration. The quenching of free radicals is 10.52% and 14.01% for the standard substance and the tested extract, respectively. The result obtained for the extract indicates the presence of other compounds in the sample that also show antioxidant activity; for example, apiin [17].

### 3.2. Microneedles

#### 3.2.1. Microneedles Patches

During the optimization of parameters to obtain the polymer base, solutions of hyaluronic acid sodium salt were prepared with concentrations of 5%, 10%, 15%, 20%, and 25% by weight. They showed different densities, namely, the higher the weight concentration of sodium hyaluronate, the denser the solution. The solution with 5 wt.% of hyaluronic acid sodium salt was very flowable so that when applied, it easily poured out of the mold (Figure S2 in the Supplementary Materials). Solutions of 10 wt.% and 15 wt.% showed the most optimal density (Figure S3 and Figure S4 in the Supplementary Materials), which allowed for convenient filling of the molds without flowing out. Solutions with concentrations of 20 wt.% and 25 wt.% (Figure S5 and Figure S6 in the Supplementary Materials) were very thick and difficult to transfer from the vial to the mold. Due to the similarities between the 10 wt.% and 15 wt.% solutions, a lower concentration, viz. 10 wt.% of hyaluronic acid sodium salt, was chosen to reduce the cost of preparing the microneedle patches.

The next step was to select the appropriate parameters to allow proper preparation and degassing of the sodium hyaluronate solution, followed by efficient mold filling. The most efficient method to prepare and degas the 10 wt.% hyaluronic acid sodium salt solution was a combination of manual stirring (approximately 2 min) and sonication (37 kHz; 25 min). The tests performed showed that the optimal option for effective mold filling was to use the freeze–thaw method.

**Figure 8:**
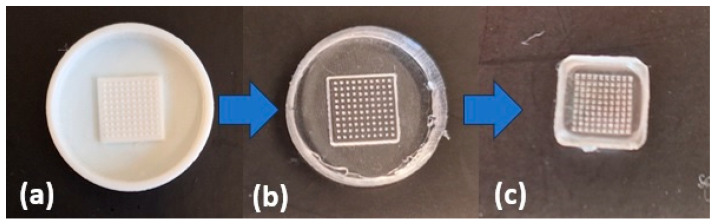
Steps to obtain the targeted microneedle patch: (**a**) polymer mold; (**b**) silicone mold; (**c**) ready microneedle patch based on sodium salt of hyaluronic acid.

Based on polymer molds, silicone molds were prepared and used to obtain microneedle patches containing selected plant extracts. The scheme to synthesize microneedle patches is shown in Figure 8.

#### 3.2.2. Stereoscopic Microscope

**Figure 9:**
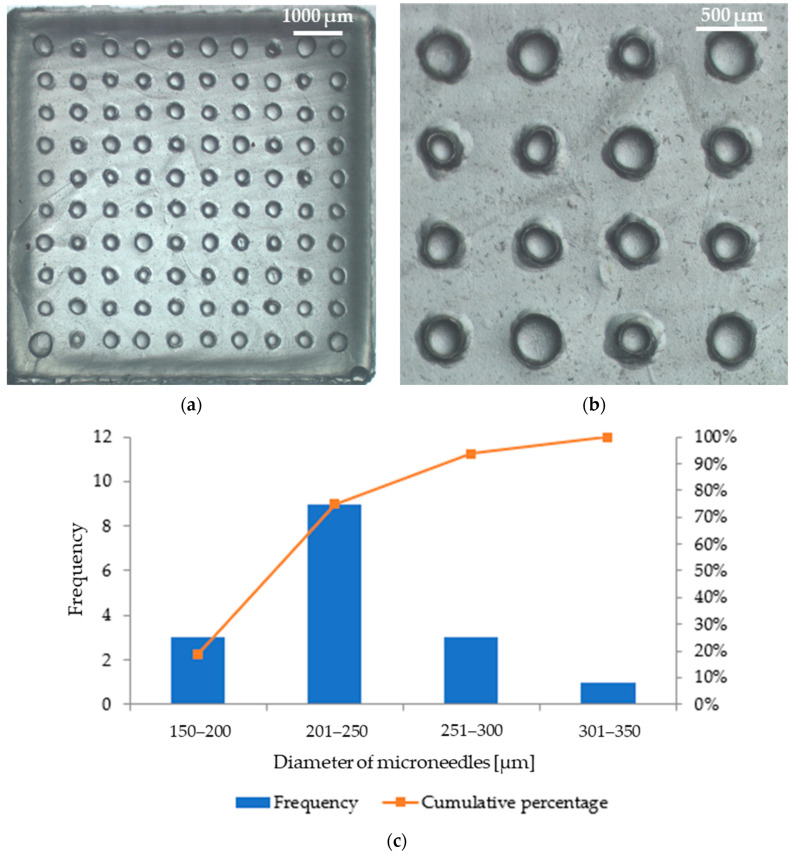
Photographs of the microneedle patch prepared with parsley leaf extract: (**a**) a view of the whole patch; (**b**) a zoom showing the arrangement of needles in the patch; (**c**) a histogram showing the frequency of individual microneedle diameters (based on Figure 9b).

**Figure 10:**
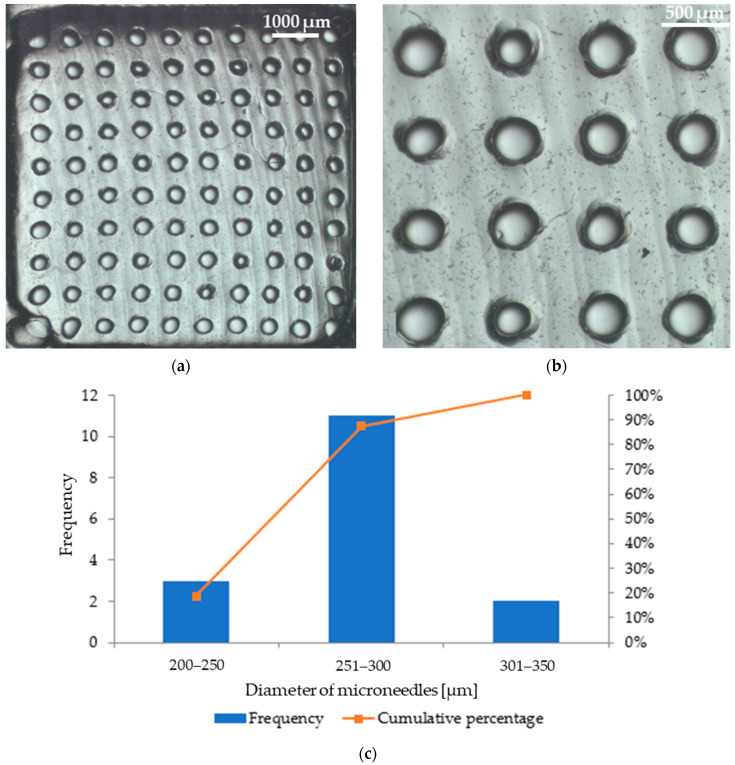
Photographs of the microneedle patch prepared with red beet extract: (**a**) a view of the whole patch; (**b**) a zoom showing the arrangement of needles in the patch; (**c**) a histogram showing the frequency of individual microneedle diameters (based on Figure 10b).

Microscopic analysis allowed us to obtain images of patches with microneedles, which are shown in Figure 9 (patch with parsley leaf extract) and Figure 10 (patch with red beet extract). The exact arrangement of the needles can be seen according to the dimensions of the polymer mold used. The needles at the base are circular in shape and have a similar diameter. Based on Figure 9b, the average diameter of the microneedles in the patch containing parsley leaf extract is 247 μm ± 42 μm. Figure 9c shows a histogram that presents the range of microneedle sizes in the patch. It can be observed that the dominant fraction of microneedles has a size between 201 μm and 250 μm. On the other hand, the average diameter of microneedles in the patch with red beet extract is 280 μm ± 32 μm (Figure 10b). The example histogram presented in Figure 10c shows that the main fraction of microneedles has a size between 251 μm and 300 μm.

**Figure 11:**
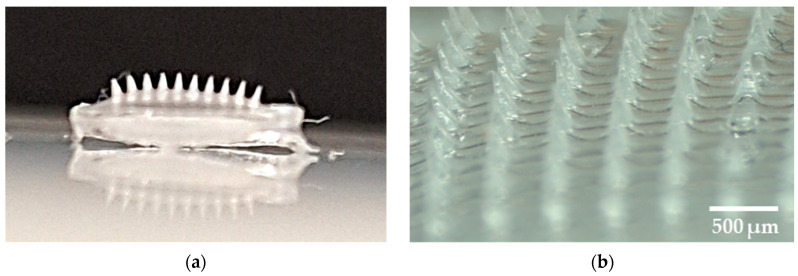
(**a**) photo showing the microneedles visible on the prepared patch; (**b**) a zoom showing the prepared patch with microneedles.

The needles formed are visible to the naked eye, as shown in Figure 11a. It can be seen that the spears are of similar length and have sharp tips. Figure 11b shows the microscopic image of the patch, depicting the alignment, length, and shape of the needles.

#### 3.2.3. Release of the Active Substance from the Microneedle Patches

**Figure 12:**
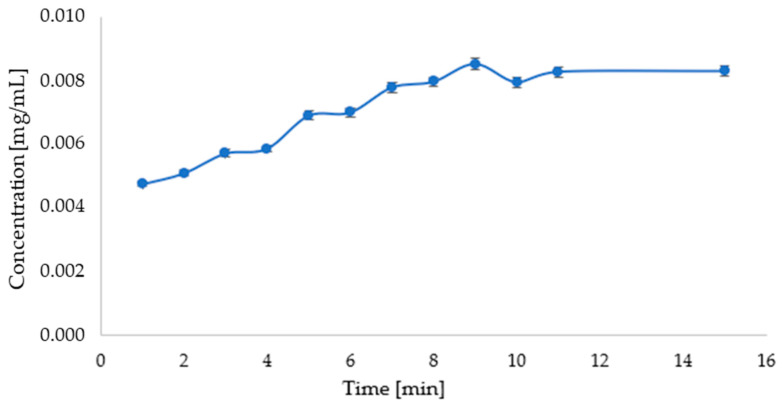
Graph showing the change in apigenin concentration over time during release into solution of phosphate buffer at pH = 5.8 (λ_max_ = 268 nm).

An analysis of the release of active substances from the prepared carriers showed progressive changes in concentration with time. In the case of the carrier with the parsley leaf extract, an increase in the concentration of the released apigenin can be observed during the first 9 min. After this time, the concentration of apigenin in the solution was constant, as shown in Figure 12.

**Figure 13:**
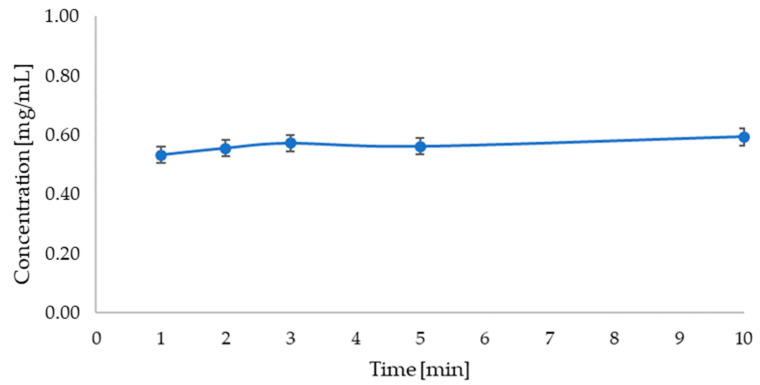
Graph showing the change in betanin concentration over time during release into solution of phosphate buffer at pH = 5.8 (λ_max_ = 536 nm).

Based on the analysis of betanin release from the carrier with the red beet extract (Figure 13), it can be observed that the betanin concentration does not change significantly during the analysis and remains at a similar level.

## 4. Discussion

### 4.1. Extracts

#### 4.1.1. Red Beet

UV–Vis spectra allowed qualitative analysis of the extracts obtained. Based on our analysis and data from the literature, it was determined that betanin showed maximum absorption at 538 nm. Therefore, it was confirmed that this active ingredient is present in both samples of the extracts. The FTIR-ATR analysis also confirmed the presence of bonds characteristic to betanin in all samples tested. LC–MS analysis confirmed the presence of betanin in the extract samples tested. According to the data from the literature, it can be concluded that it is possible to confirm the content of betanin and other compounds from betalanin group on the basis of LC–MS analysis. Based on the results described earlier in the literature by Wybraniec et al., betanin and other pigments found in red beetroot can also be determined using this technique [39,40]. Chromatographic analyses, in turn, enabled the selection of the most efficient extraction method for the plant raw materials used. The extract obtained after 5 h of the ultrasound-assisted procedure was characterized by the highest abundance of compounds, including betanin, which exhibits anti-inflammatory, antioxidant, and anticancer properties, which was also confirmed by studies previously reported in the literature [34,41].

Maceration for 24 h allowed us to obtain similar results from the GC–MS analysis as in the ultrasound-assisted method. However, the duration of this extraction is long and thus requires a higher energy input. On the other hand, 5 h of maceration did not give the expected results, as due to the shorter extraction time most of the compounds were not isolated, and their signals on the GC–MS spectra were traceable. The differences in the performance of ultrasound-assisted extraction and maceration may be due to the way ultrasound works. During maceration, the compounds are moved to the solution through diffusion, while ultrasound causes wave-like pressure changes to accelerate extraction, making it more efficient [42,43]. Nevertheless, according to the literature data, it should be noted that the qualitative composition of the red beetroot extract may depend not only on the method of extraction of the sample but also on the place of cultivation. Depending on soil fertility, atmospheric conditions, and the amount of rainfall or fertilizers used, the qualitative composition of the extract can be different, since all of these factors affect the cultivation of this plant and the compounds it takes from the soil [44]. Testing antioxidant activity against the DPPH radical allowed us to confirm the properties of these active substances as well as the tested extract. Thus, it can be assumed that red beet extract can have antioxidant properties on the skin.

#### 4.1.2. Parsley Leaves

UV–Vis spectra allowed for the qualitative analysis of the extracts under study. On the basis of the obtained results of the analysis and literature data, it was observed that apigenin exhibits two absorption maxima at wavelengths of 339 nm and 269 nm. On this basis, it was confirmed that both extract samples contain this active substance. FTIR-ATR analysis also manifested the characteristic bonds in all tested samples, confirming the presence of apigenin. During LC–MS analysis, on the basis of the retention time of the standard of the active compound, the presence of apigenin in the extracts obtained was confirmed. GC–MS analysis allowed the identification of a number of compounds present in the extracts obtained. Staropoli et al. also subjected parsley leaves in powdered form to LC–MS and GC–MS analysis, during which large amounts of apiole, a compound that was also isolated in our samples, were identified [45]. In the extract obtained using the ultrasound-assisted method, fewer compounds or those with a trace signal were identified in GC-MS analysis, compared to the samples of extracts prepared during 5 h and 24 h of maceration. Therefore, it can be concluded that the best extraction method for parsley is maceration. Based on the literature data, it can be stated that the qualitative composition of parsley extract can depend on the type of extraction method applied, as well as the place of cultivation. Depending on soil fertility or general atmospheric conditions, plants can take up different substances from the soil. Furthermore, the amount of sunlight reaching the plant can affect its development and chemical composition [46,47]. A longer maceration time (24 h) allowed for the identification of a slightly larger number of compounds, which is why it can be considered the best extraction procedure. The slight difference in the number of identified compounds may be due to the fundamentals of the selected extraction methods. Maceration involves the diffusion of compounds from the plant material into the extract, while ultrasound-assisted extraction generates pressure changes that can affect extraction efficiency [19,48]. Testing antioxidant activity against the DPPH radical allowed us to confirm the properties of these active substance as well as the tested extract. Therefore, it can be assumed that it will also exhibit such an effect when applied to the skin.

### 4.2. Microneedles

According to our results, the most optimal concentration of the solution of hyaluronic acid sodium salt is 10 wt.%. This solution can be conveniently applied to the mold and does not spill out of it. The best method for the dissolution and degassing of the sodium hyaluronate solution is a combination of manual stirring for approximately 2 min and sonication (37 kHz) for approximately 25 min. The polymer mold was used to prepare a silicone mold, into which the solution of sodium hyaluronate with the plant extract was poured. The freezing–thawing procedure allowed the most precise filling of the silicone mold and the elimination of air bubbles. The 10 wt.% solution of hyaluronic acid sodium salt together with 1 wt.% of the plant extract had the appropriate consistency for easy and convenient filling of the molds and allowed the distribution of the extracts throughout the volume of the patch mold with microneedles. Left in a silicone mold for 24 h at room temperature, the solution evaporated, transforming into a hard but not brittle patch that could be easily separated from the mold. The images from the stereoscopic microscope show that the resulting needles have the desired length and shape. We can observe that the diameter of the microneedles in the patches containing plant extracts differs and ranges from 200 μm to 300 μm. It can also be observed that the diameters of the produced microneedles differ slightly from the dimensions of the polymer mold. The reason for this may be the insufficient accuracy of the polymer mold and silicone mold used to obtain the actual microneedle patches.

A release study of the active ingredient from the carrier with beet extract in a pH = 5.8 phosphate-buffered solution suggests that betanin release from the carrier occurs very quickly. However, the release study of the active ingredient from the carrier with the parsley leaf extract suggests that the release of apigenin from the carrier occurs gradually up to about 9 min and then remains constant.

## 5. Conclusions

The extraction methods presented allow for obtaining extracts containing numerous active substances, including those with antioxidant, anti-inflammatory, and anticancer properties. The best procedure for the extraction of red beetroot turned out to be the ultrasound-assisted method, while the best parsley leaf extract turned out to be the one obtained after 24 h of maceration. The extracts were successfully introduced into modern transdermal carriers, namely microneedle patches. The soluble patches obtained, with microneedles based on the sodium salt of hyaluronic acid and containing beetroot or parsley extracts, can be applied directly to inflammatory skin changes, delivering active compounds to the deeper layers of the skin.

## Figures and Tables

**Table 1:** Comparison of compounds identified in different red beet extract samples on the basis of GC–MS spectra.

Compound	Retention Time [min]	M_5H	M_24H	U_5H
Pyrimidine	2.470	s	+	+
DL-Arabinose	3.421	+	+	+
1H-Imidazole, 4,5-dihydro-2-methyl	4.970	+	+	+
DL-Pantolactone	6.509	+	+	+
Maltol	6.852	s	+	+
Lysidine	7.411	s	+	+
Pyranone	9.030	+	+	+
5-Hydroxymethylfurfural	12.450	−	+	+
Butanoic acid	12.807	s	+	+
Heptanoic acid	14.430	s	+	+
2-Methoxy-4-vinylphenol	15.550	−	+	+
Decanedioic acid	18.027	−	+	+
Sucrose	21.734	+	+	+
7,9-Di-tert-butyl-1-oxaspiro(4,5)deca-6,9-diene-2,8-dione	47.373	+	+	+
n-Hexadecanoic acid	50.577	+	+	+
9-Octadecenamide, (Z)	52.603	+	-	+

(s) Trace signal, (+) Presence of the compound in the tested sample, (−) Not identified.

**Table 2:** Comparison of compounds identified in different parsley leaf extract samples on the basis of GC–MS spectra.

Compound	Retention Time [min]	M_5H	M_24H	U_5H
Glyceraldehyde	2.391	+	+	s
Butanoic acid	2.856	+	+	s
1,2,3,4-Butanetetrol, [S-(R,R)]	3.099	+	+	s
1,2-Cyclopentanedione	3.562	+	+	+
α-Pinene	3.669	s	s	+
Glycerin	4.235	+	+	-
β-Myrcene	4.561	+	+	+
β-Phellandrene	5.635	+	+	+
Maltol	6.847	+	+	−
p-Cymenene	7.276	+	+	+
Ethyl hydrogen malonate	7.465	+	+	s
p-Mentha-1,5,8-triene	7.977	+	+	+
Pyranone	9.028	+	+	s
2-Naphthol, 1,2,3,4,4a,5,6,7-octahydro-4a-methyl	10.729	+	+	+
1-Deoxy-d-arabitol	11.026	+	+	+
8,9-Dehydrothymol	11.666	+	+	+
trans-p-mentha-1(7),8-dien-2-ol	12.645	−	+	−
1,2,3-Propanetriol, 1-acetate	12.792	+	+	s
(+)-Diethyl L-tartrate	12.986	+	+	s
Pentanoic acid	13.552	+	+	s
Methyl 6-oxoheptanoate	14.480	+	+	+
Caryophyllene	18.565	+	+	+
Sucrose	19.828	+	+	+
2,4-Di-tert-butylphenol	21.846	+	+	+
Myristicin	22.462	+	+	+
1,2,3,5-Cyclohexanetetrol, (1α,2β,3α,5β)	28.532	+	+	+
Apiol	32.598	+	+	+
Neophytadiene	44.788	+	+	+
Phytol	44.924	+	+	+
Acetamide, N-(2-phenylethyl)	46.267	+	+	+
7,9-Di-tert-butyl-1-oxaspiro(4,5)deca-6,9-diene-2,8-dione	47.369	+	+	s
Dibutyl phthalate	49.862	−	+	−
n-Hexadecanoic acid	50.595	+	+	+
9-Octadecenamide, (Z)	53.518	+	+	s

(s) Trace signal, (+) Presence of the compound in the tested sample, (−) Not identified.

## Data Availability

Dataset available on request from the authors.

## Supplementary Materials

The following supporting information can be downloaded at: https://www.mdpi.com/article/10.3390/mi16020143/s1, Figure S1: “Substitute” silicone mold in the form of a square, which was used to simulate the target mold; Figure S2: Stereoscopic image of a 5 wt.% solution of sodium salt of hyaluronic acid after removal from the mold; (a) the whole sample; (b) in zoom; Figure S3: Stereoscopic image of a 10 wt.% solution of sodium salt of hyaluronic acid after removal from the mold; (a) the whole sample; (b) in zoom; Figure S4: Stereoscopic image of a 15 wt.% solution of sodium salt of hyaluronic acid after removal from the mold; (a) the whole sample; (b) in zoom; Figure S5: Stereoscopic image of a 20 wt.% solution of sodium salt of hyaluronic acid after removal from the mold; (a) the whole sample; (b) in zoom; Figure S6: Stereoscopic image of a 25 wt.% solution of sodium salt of hyaluronic acid after removal from the mold; (a) the whole sample; (b) in zoom; Figure S7: Standard curve for betanin (λ_max_ = 536 nm); Figure S8: Standard curve for apigenin (λ_max_ = 268 nm); Figure S9: Change in absorbance of the DPPH radical solution due to reaction with (a) standard substance - betanin; (b) red beet extract obtained with ultrasound (B_U_5h); Figure S10: Change in absorbance of the DPPH radical solution due to reaction with (a) standard substance - apigenin; (b) parsley leaves extract obtained with 24 h maceration (P_M_24h).
