# Supplementary material for: Microneedles as Modern Carriers of Plant Extracts
Source: Micromachines (Basel). 2025 Jan 26;16(2):143. doi: 10.3390/mi16020143 (PMC11857659; doi:10.3390/mi16020143)
Supplement: Supplementary file 1 [file micromachines-16-00143-s001.zip › micromachines-3415324-supplementary.pdf]

SUPPLEMENTARY MATERIALS

Microneedles as modern carriers of plant extracts

Jagoda Chudzińska-Skorupinska [1], Agata Wawrzyńczak [2] and Agnieszka Feliczak-Guzik [3]*

[1] Adam Mickiewicz University in Poznań, Faculty of Chemistry, Department of Applied Chemistry; Uniwersytetu Poznańskiego 8, 61-614 Poznań, Poland; jagoda.chudzinska@amu.edu.pl
[2] Adam Mickiewicz University in Poznań, Faculty of Chemistry, Department of Applied Chemistry; Uniwersytetu Poznańskiego 8, 61-614 Poznań, Poland; agata.wawrzynczak@amu.edu.pl
[3] Adam Mickiewicz University in Poznań, Faculty of Chemistry, Department of Applied Chemistry; Uniwersytetu Poznańskiego 8, 61-614 Poznań, Poland; agnieszka.feliczak-guzik@amu.edu.pl
* Correspondence: agnieszka.feliczak-guzik@amu.edu.pl

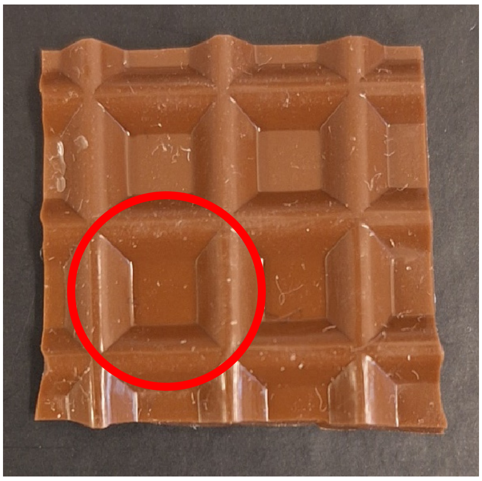

Figure S1. “Substitute” silicone mold in the form of a square, which was used to simulate the target mold.

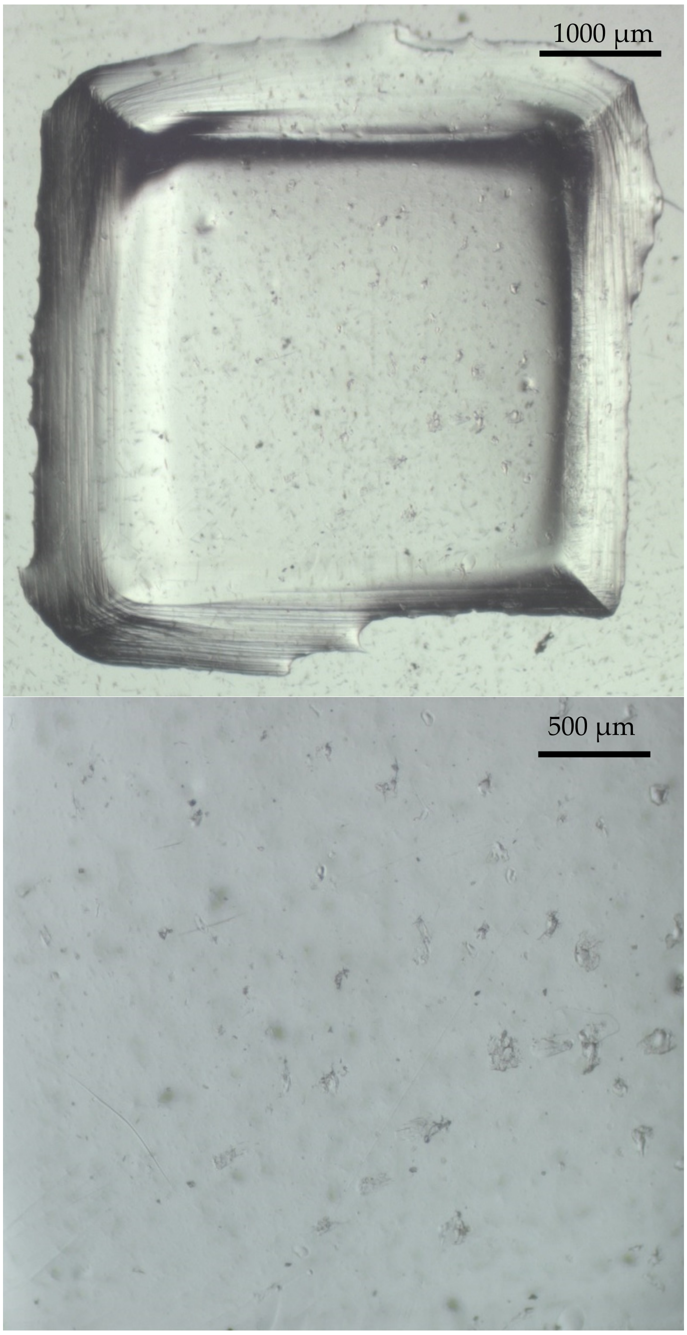

Figure S2. Stereoscopic image of a 5 wt.% solution of sodium salt of hyaluronic acid after removal from the mold; (a) the whole sample; (b) in zoom.

(a)

(b)

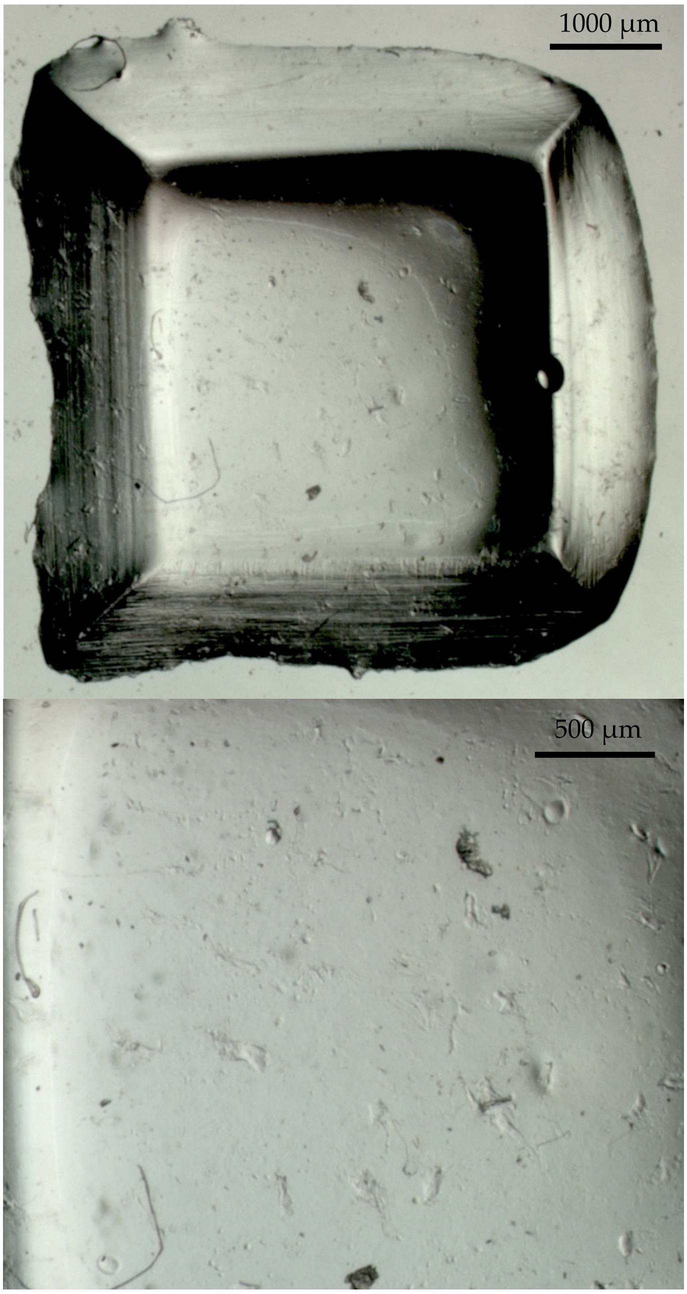

Figure S3. Stereoscopic image of a 10 wt.% solution of sodium salt of hyaluronic acid after removal from the mold; (a) the whole sample; (b) in zoom.

(a)

(b)

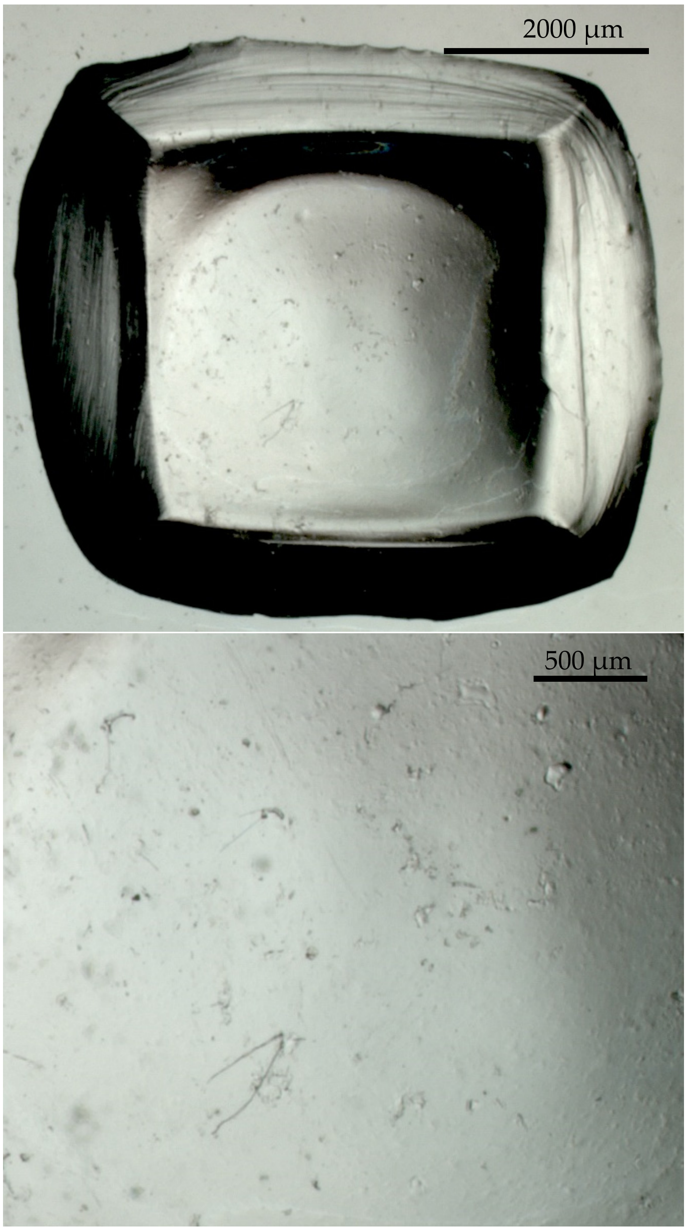

Figure S4. Stereoscopic image of a 15 wt.% solution of sodium salt of hyaluronic acid after removal from the mold; (a) the whole sample; (b) in zoom.

(a)

(b)

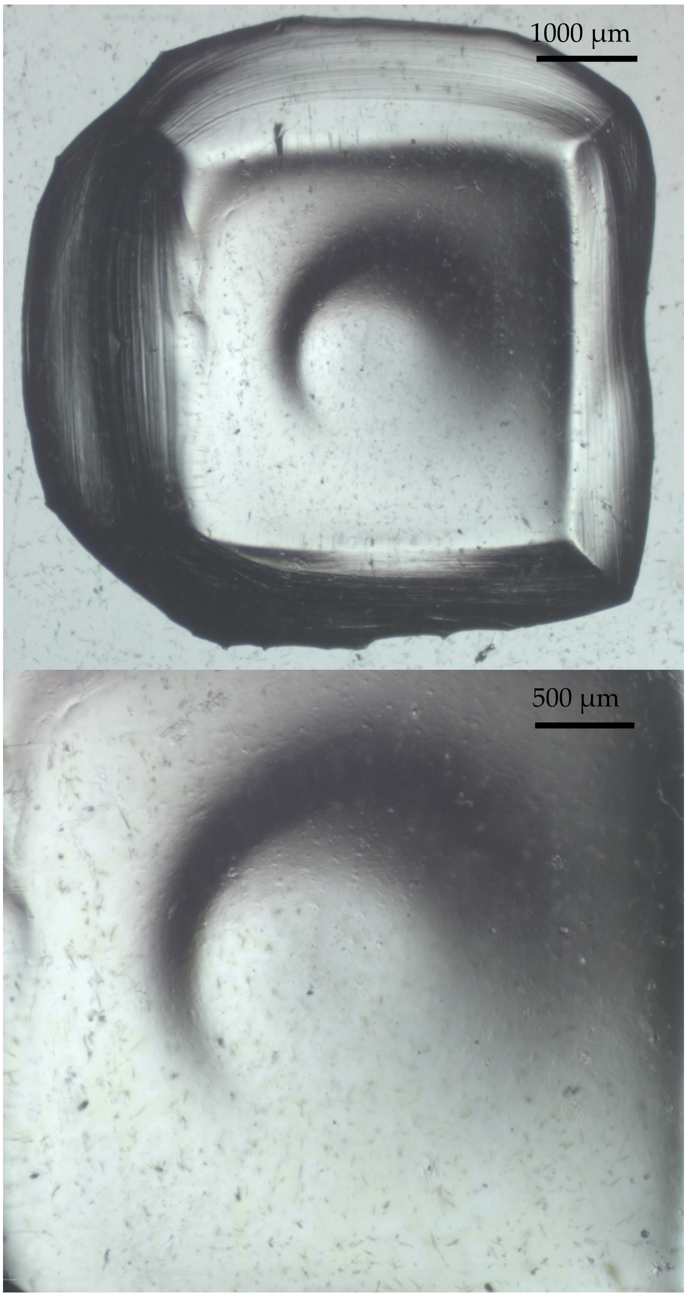

Figure S5. Stereoscopic image of a 20 wt.% solution of sodium salt of hyaluronic acid after removal from the mold; (a) the whole sample; (b) in zoom.

(a)

(b)

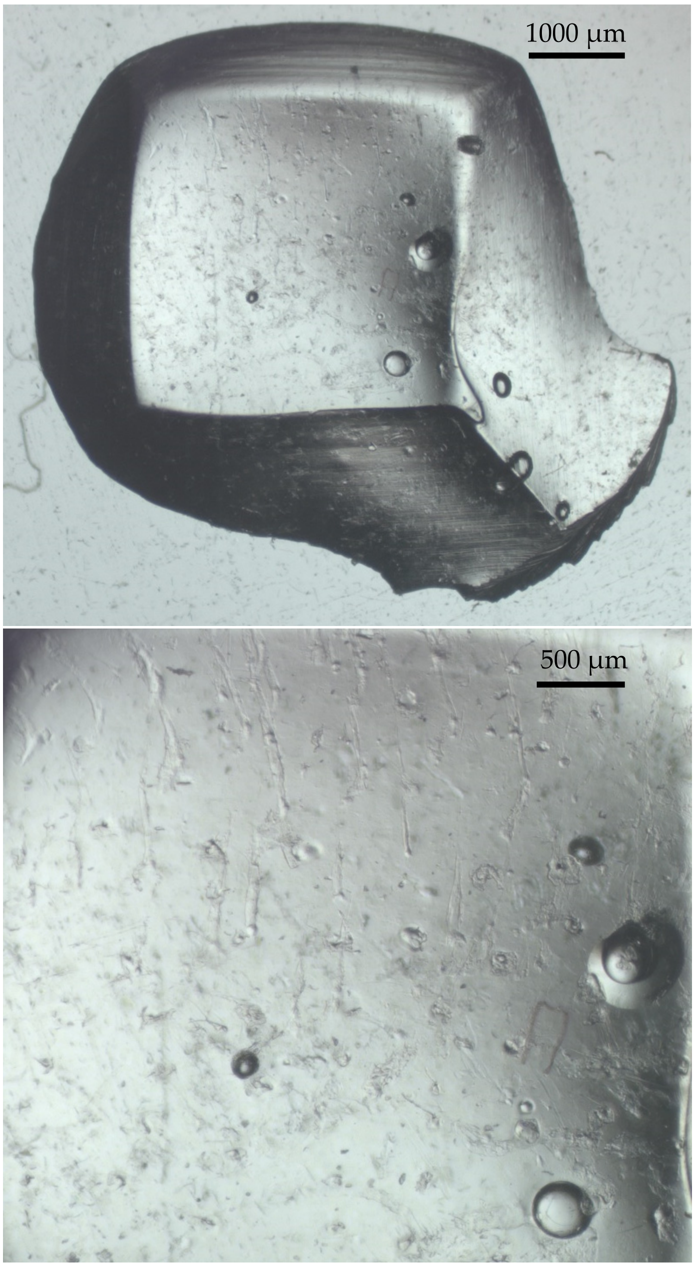

Figure S6. Stereoscopic image of a 25 wt.% solution of sodium salt of hyaluronic acid after removal from the mold; (a) the whole sample; (b) in zoom.

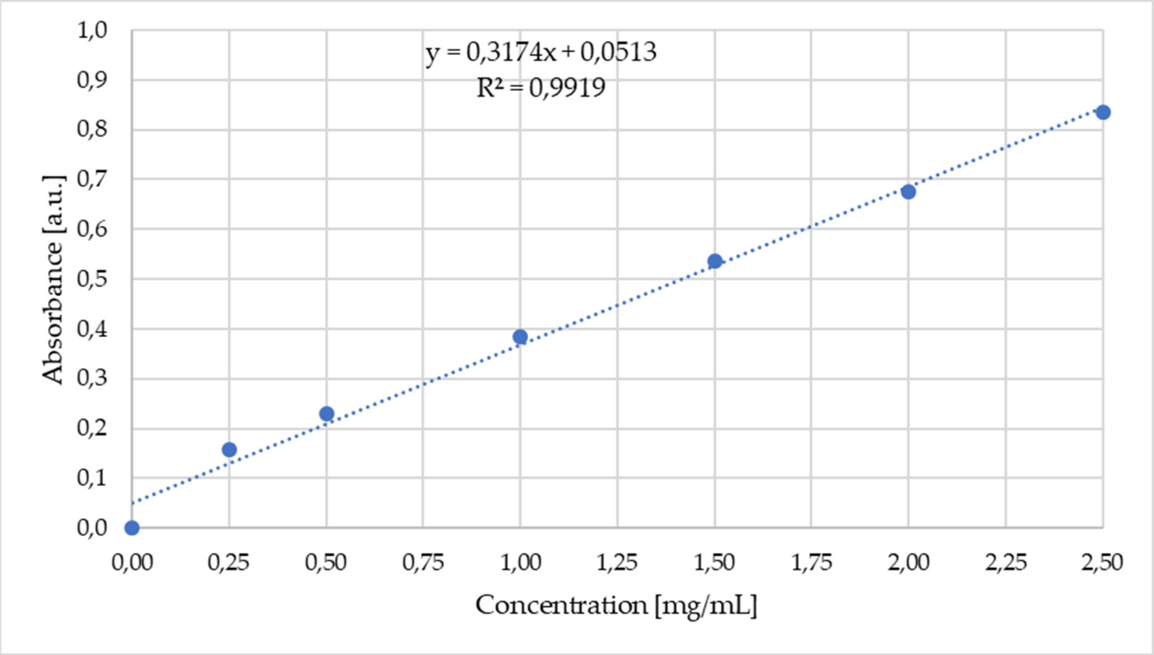

Figure S7. Standard curve for betanin (λ_{max} = 536 nm).

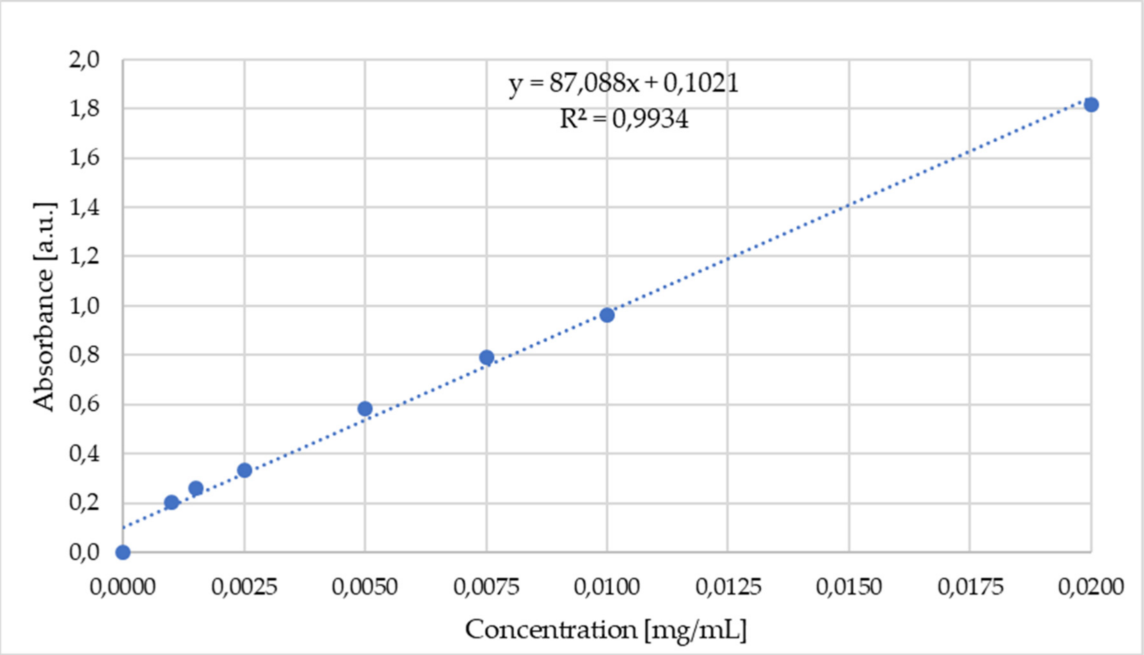

Figure S8. Standard curve for apigenin (λ_{max} = 268 nm).

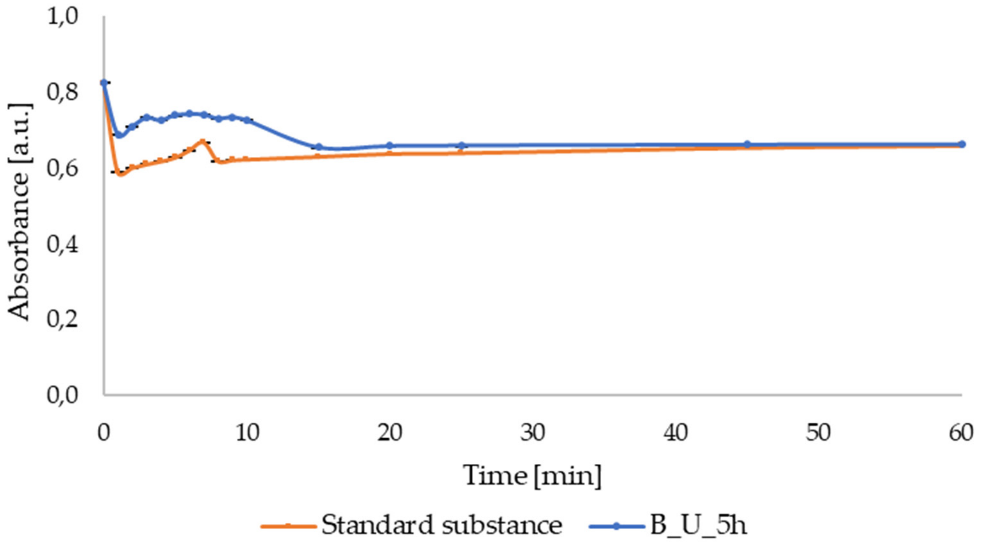

Figure S9. Change in absorbance of the DPPH radical solution due to reaction with (a) standard substance - betanin; (b) red beet extract obtained with ultrasound (B_U_5h).

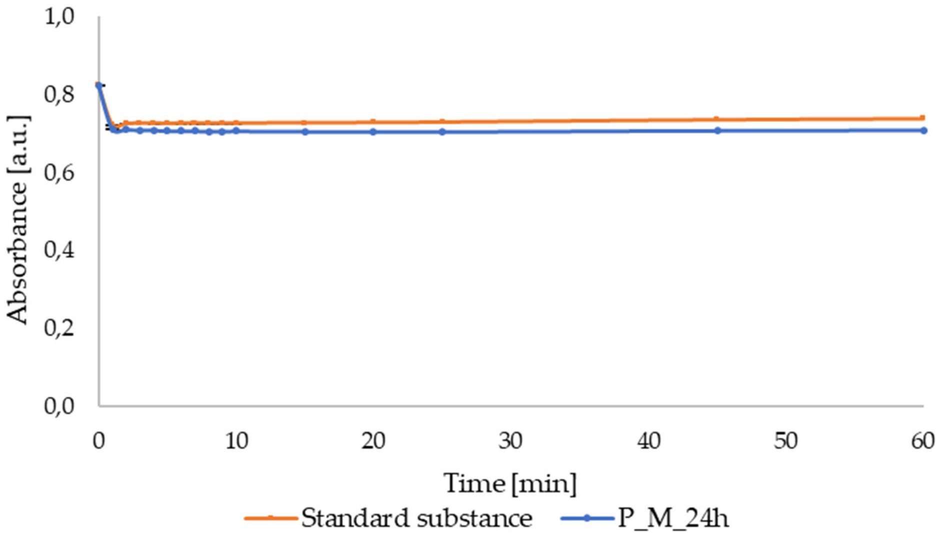

Figure S10. Change in absorbance of the DPPH radical solution due to reaction with (a) standard substance - apigenin; (b) parsley leaves extract obtained with 24h maceration (P_M_24h).
